# Effect of vitamin E supplementation on uterine cervical neoplasm: A meta-analysis of case-control studies

**DOI:** 10.1371/journal.pone.0183395

**Published:** 2017-08-22

**Authors:** Xiaoli Hu, Saisai Li, Lulu Zhou, Menghuang Zhao, Xueqiong Zhu

**Affiliations:** Department of Obstetrics and Gynecology, the Second Affiliated Hospital and Yuying Children’s Hospital of Wenzhou Medical University, Wenzhou, China; University of Texas Health Science Center, UNITED STATES

## Abstract

Several epidemiological studies have suggested that vitamin E could reduce the risk of uterine cervical neoplasm. However, controversial data were presented by different reports. Hence, we conducted a meta-analysis to assess the relationship between vitamin E and the risk of cervical neoplasia. We performed a comprehensive search of the PubMed, Embase and Cochrane databases through December 31, 2016. Based on a fixed-effects or random-effects model, the odds ratio (OR) and 95% confidence intervals (CIs) were calculated to assess the combined risk. Subgroup analyses and meta-regression were done to assess the source of heterogeneity. Subgroup analyses were performed according to survey ways, types of cervical neoplasia, study populations. A protocol was registered with PROSPERO (No. CRD42016036672). In total, 15 case-control studies were included, involving 3741 cases and 6328 controls. Our study suggested that higher category of vitamin E could reduce the cervical neoplasia risk (OR = 0.58, 95% CIs = 0.47–0.72, *I*^2^ = 83%). In subgroup-analysis, both vitamin E intake and blood levels of vitamin E had a significant inverse association with the risk of cervical neoplasm. Additionally, we found the same relationship between vitamin E and cervical neoplasia among different populations and types of cervical neoplasia. Meta-regression showed that none of the including covariates were significantly related to the outcomes. No evidence of publication bias was observed. In conclusion, vitamin E intake and blood vitamin E levels were inversely associated with the risk of cervical neoplasia.

## Introduction

Cervical cancer remains the third most common gynecological malignancy in the world and the leading cause of mortality among women in developing countries [1], and responsible for an estimated 265,000 deaths annually worldwide, 87% occurring in low-resource countries [2]. Overwhelming evidence now supports the role of human papillomaviruses (HPV) in cervical carcinogenesis [3–4]. However, it has generally been acknowledged that lifestyle factors may play an important role in the prevention of cervical carcinoma [5], and many studies have shown that dietary vitamin E might reduce the risk of cervical dysplasia and cancer [6–7].

Vitamin E, which contains putative anticancer and antimutagenic substances, had long been thought to protect against cancer, including cervical cancer. Vitamin E is a well-known inhibitor of lipid peroxidation and a powerful antioxidant, which has been reported to protect cells from oxidative DNA damage and mutagenesis, thereby preventing the occurrence of some tumors [8–9].

In past few years, a number of epidemiology studies have been conducted to assess the relationship between dietary vitamin E and cervical neoplasia which included both cervical cancer and cervical intraepithelial neoplasia (CIN), but the results were not consistent [6,10–23]. To date, many researches revealed that vitamin E was associated with a decreased risk of cervical neoplasia [6,11,18]; however, the other studies suggested that there was no connection between vitamin E and the decreased risk of cervical neoplasia [12]. Therefore, we conducted a meta-analysis in order to better evaluate the association between vitamin E and the risk of cervical neoplasia by combining the results from the published observational studies.

## Method

### Search strategy

The review met requirements of the Preferred Reporting Items for Systematic reviews and Meta-Analyses (PRISMA) statement. A protocol of this meta-analysis was registered in PROSPERO International prospective register of systematic reviews (registration number: CRD42016036672). We searched the PubMed, Embase and Cochrane library databases from their inception. The first search was conducted on July 28, 2016 and the latest search on December 31, 2016. The following keywords were used in searching: (“uterine cervical neoplasms” or “cervical cancer” or “cervical tumor” or “cervical malignance” or “cervical carcinoma” or “cervical neoplasm” or “cervical intraepithelial neoplasia”) and (“vitamin E” or “Vit E” or “tocopherols” or “alpha-tocopherol” or “antioxidant” or “diet”) with no restrictions. S1 Table provided specific details on the search terms in PubMed database and strategy used to collect the records for screening from both searches employed. A manual search of references cited in the selected articles and published reviews were also performed for additional studies. The study protocol was approved by the Research Ethical Committee of the Second Affiliated Hospital and Yuying Children’s Hospital of Wenzhou Medical University.

### Study selection

Relevant studies were selected by two independent reviewers (XLH, MHZ), and any disagreement was resolved by discussion. The studies which met the following criteria could be included: (1) the study had a case-control or prospective cohort designed; (2) reported the association between vitamin E intake or blood levels of vitamin E and the risk of cervical neoplasia (CN), including invasive cervical carcinoma, cervical dysplasia, in situ cervical cancer, cervical intraepithelial neoplasia (CIN) and total cervical cancer; (3) papers were restricted to human studies published as full-length articles in English; (4) the odds ratio (OR) estimates with the corresponding 95% confidence intervals (CIs) (or data to calculate these) for the highest vs. the lowest level of vitamin E intake or blood levels of vitamin E were reported. Accordingly, studies were excluded if (1) they were reviews, letters, animal experiments, or comments; (2) they were duplicate publications; (3) they were not published as a full text; (4) the OR with 95% CIs were not usable.

### Data extraction and quality assessment

The following information were extracted from the included studies: the first author’ last name, year of publication, study population, number of cases and controls, age range of study participants, study quality, OR (95% CIs) from the most fully adjusted model for the highest compared with the lowest dietary vitamin E intake or blood levels of vitamin E, adjusted covariates. If there were disagreements between the two authors about eligibility of the data, they were resolved by consensus. If one study included several kinds of vitamin E or cervical neoplasia, we defined the research as -1, -2 and so on. The study quality was assessed by using the 9-star Newcastle-Ottawa Scale (NOS) [24]. This scale was categorized into 8 items: adequate definition of cases such as a histological diagnosis of CIN or cervical cancer; representativeness of the cases; selection of controls; definition of controls; control for important factor or additional factor like ages (two stars); ascertainment of exposure such as the questionnaire; same method to ascertain for cases and controls; non-response rate, with a total of 9 stars. This scale was a risk of bias assessment tool for observational studies, especially case-control or cohort studies. It was recommended by the Cochrane Collaboration. However, this assessment tool was lack of methodological details in published studies, which may potentially deviate the risk of bias assessment. According to the scoring system, the study quality was defined as high with scores no less than 7 and as moderate with scores of 4–6, while as inferior with scores no more than 3.

### Statistical analysis

Statistical analyses were performed by RevMan5.3 software (The Nordic Cochrane Center). Besides, STATA software (version 12.0; Stata Corporation, College Station, TX) was used to perform the Egger’s test and Begg’s test which could detect publication bias. Heterogeneity test was conducted by using Q and *I*^*2*^ statistics [25]. Additionally, *I*^*2*^ values of 0, 25, 50 and 75% represented no, low, moderate and high heterogeneity, respectively. A fixed-effects model was used to calculate the pooled OR when the heterogeneity of each study was low or *P* value of heterogeneity > 0.1. Otherwise, a random-effects model was used for outcomes when strong evidence of heterogeneity was found (*P*_heterogeneity_ ≤ 0.10, or *I*^*2*^ > 50%). OR and 95% CIs were calculated to assess the association between vitamin E intake or blood levels of vitamin E and the risk of cervical neoplasia.

Meanwhile, subgroup analysis was used to investigate the possible source of heterogeneity among these included studies. Subgroup analyses were performed to according to survey ways, types of cervical neoplasia, geographical regions. Also, possible sources of heterogeneity were indicated by meta-regression. In order to assess the statistical outcome validity, we detected overall outcome by sensitivity analysis.

In addition, a p-value < 0.05 in the Egger’s test or Begg’s test was considered as statistically significant for publication bias. We used the funnel plot to detect the publication bias [26].

## Results

### Literature search

**Fig 1:**
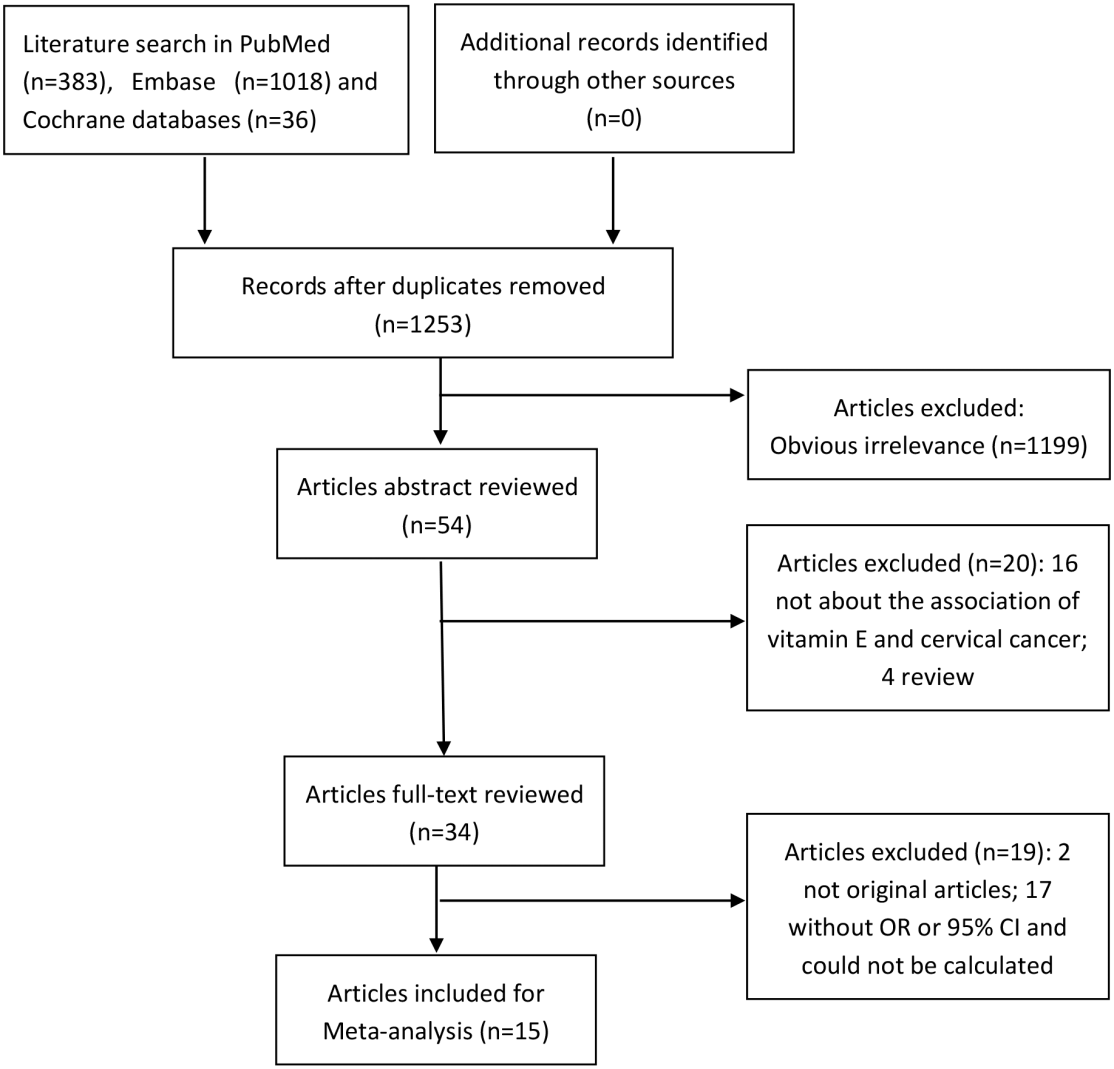
Flow chart of literature selection.

By the search strategy, we identified a total of 1253 studies (383 from PubMed, 1018 from Embase, 36 from Cochrane databases) after deleting the duplicate researches. After excluding researches that studied obvious irrelevant exposure/outcome, or failed to report OR or 95% CIs, or didn’t meet the inclusion criteria, we identified a total of 15 observational studies involving 3741 cases and 6328 controls [6,10–23]. These studies were published between 1990 and 2015. Moreover, these studies included 6 on vitamin E intake and 10 on blood vitamin E levels. The study selection process was presented in Fig 1.

### Study characteristics

The main characteristics of the 15 studies are given in Table 1. The studies were published from 1990 to 2015. Meanwhile, the cancer types included 11 studies about cervical cancer, 10 researches about CIN. There was no randomized controlled trial (RCT) or cohort study included in our meta-analysis based on the criteria. All the ORs in the 15 studies were estimated based on the highest compared with the lowest dietary vitamin E intake or blood levels of vitamin E. Of those studies, 9 studies were conducted in America [6,10,13–16,18,22,23], 5 in Asian populations [11–12,17,19,21], 1 study in Europe [20]. All the studies were adjusted for a wide range of potential confounders, such as age, education, parity, and HPV infection status.

**Table 1:** Main characteristics of included studies.

Study (year, population)	Age of subjects	Sample size (n) case/ controls (total)	Type of CN	Type of Vitamin E	OR (95% CIs)	Study Quality	Adjustment for Covariates
Gloria et al. (1998, American)	NR	378/366	CIN	α-tocopherol-plasma	0.47 (0.29–0.75)	7	HPV positivity, age, ethnicity, annual household income and current smoking status.
Kim et al. (2010, Korea)	20–75	144/288	cervical cancer	vitamin E-intake	0.54 (0.30–0.99)	8	age, smoking status, alcohol consumption status, exercise, family history, body mass index, and human papillomavirus infection status.
Nagata et al. (1999, Japan)	≤55	167/167	cervical dysplasia	α-tocopherol-serum	0.80 (0.46–1.40)	6	HPV infection and smoking.
Potischman et al. (1991, America)	NR	387/670	invasive cervical cancer	α-tocopherol-serum	0.94 (0.65–1.34)	6	age, study site, age at first sexual intercourse, number of sexual partners, number of pregnancies, presence of human papillomavirus 16/18, interval since last cervical Papanicolaou smear, cholesterol, and triglycerides.
				γ-tocopherol-serum	1.37 (0.96–1.96)		
Tomita et al. (2010, Brazil)	21–65	605/453	CIN II	α-tocopherol-serum	0.39 (0.22–0.69)	8	age, hospital, ethnicity, education and potential confounders (smoking, sexual debut, lifetime sexual partner and parity) or mediators (HPV status) if their inclusion in any of the models caused a change in the OR estimate of 10% or more.
				γ-tocopherol-serum	0.77 (0.46–1.31)		
			CINIII	α-tocopherol-serum	0.26 (0.14–0.46)		
				γ-tocopherol-serum	0.44 (0.28–0.69)		
			invasive cervical cancer	α-tocopherol-serum	0.81 (0.45–1.43)		
Tomita et al. (2011, Brazil)	21–65	231/453	CIN III	α-tocopherol-serum	0.86 (0.54–1.38)	7	Age, hospital, race/ethnicity, potential confounders or mediators if their inclusion in any of the models caused a change in the OR estimate of 10% or more: sexual debut, lifetime sexual partner, parity and HPV status.
				γ-tocopherol-serum	0.60 (0.41–0.86)		
Yeo et al. (2000, American)	18–45	302/326	CIN I	α-tocopherol-serum	0.67 (0.40–1.13)	7	HPV status, age, annual family income, current residence, and lifetime number of sexual partners.
			CIN II/III	γ-tocopherol—serum	0.44 (0.23–0.86)		
Cho et al. (2009, Korea)	NR	484/378	CIN I	α-tocopherol-serum	0.61 (0.36–1.04)	8	age, menopause, parity, oral contraceptive, smoking status, alcohol consumption, and HPV infection status.
				γ-tocopherol-serum	0.57 (0.33–1.00)		
			CIN II/III	α-tocopherol-serum	0.22 (0.11–0.40)		
				γ-tocopherol-serum	0.47 (0.27–0.81)		
			cervical cancer	α-tocopherol-serum	0.27 (0.15–0.48)		
				γ-tocopherol-serum	0.16 (0.09–0.31)		
Goodman et al. (1998, America)	8–84	147/191	cervical dysplasia	vitamin E-serum	0.25 (0.13–0.48)	7	age, ethnicity, tobacco smoking, alcohol drinking, HPV detection by PCR dot-blot hybridization and plasma cholesterol.
				α-tocopherol-serum	0.24 (0.13–0.48)		
				γ-tocopherol-serum	1.02 (0.55–1.88)		
				&-tocopherol-serum	0.63 (0.34–1.16)		
Guo et al. (2015, China)	18–70	458/742	invasive cervical cancer	vitamin E-serum	0.50 (0.36–0.69)	8	age, body mass index (BMI), marital status, education, family history of cancers, HPV infection, passive smoking, current alcohol drinking, calcium supplement use, multivitamin use, menopause, oral contraceptive use, estrogen use, physical activity, and daily energy intake (log-transformed).
				vitamin E-intake	0.46 (0.32–0.64)		
Kwaśniewska et al. (1997, Germany)	NR	324/228	cervical dysplasia	α-tocopherol-serum	0.25 (0.16–0.41)	6	NR
Shannon et al. (2002, Thailand)	NR	184/509	invasive cervical cancer	vitamin E-intake	0.94 (0.52–1.71)	7	live births, screening chest X-ray and HPV.
			in-situ cervical cancer		0.72 (0.28–1.89)		
Wideroff et al. (1998, America)	NR	251/806	CIN	vitamin E-intake	1.37 (0.87–2.16)	7	age
Ghosh et al. (2008, America)	21–90	239/979	cervical cancer	vitamin E-intake	0.58 (0.41–0.82)	8	age, education, smoking status, oral contraceptive use, barrier and spermicide use, family history of cervical cancer, year questionnaire completed, and total energy intake.
Slattery et al. (1990, America)	20–59	266/408	cervical cancer	vitamin E-intake	0.59 (0.37–0.93)	7	age, education, cigarette smoking, church attendance, and number of sex partners.

NR, not reported; CN, cervical neoplasia; CIN, cervical intraepithelial neoplasia; OR, The odds ratio; CIs, 95% confidence intervals. Study quality was judged based on the Newcastle-Ottawa Scale (range, 1–9 stars).

### Study quality assessment

The quality assessment of studies based on NOS system was also presented in Table 1. In this review, the quality score of the includes studies ranged from 6 stars to 8 stars and 80% of the studies were identified as high-quality. Meanwhile, the mean ± standard deviation from all the NOS scores was 7.13 ± 0.74 scores which were numerically high in NOS system.

### The relationship between vitamin E and cervical neoplasia

**Fig 2:**
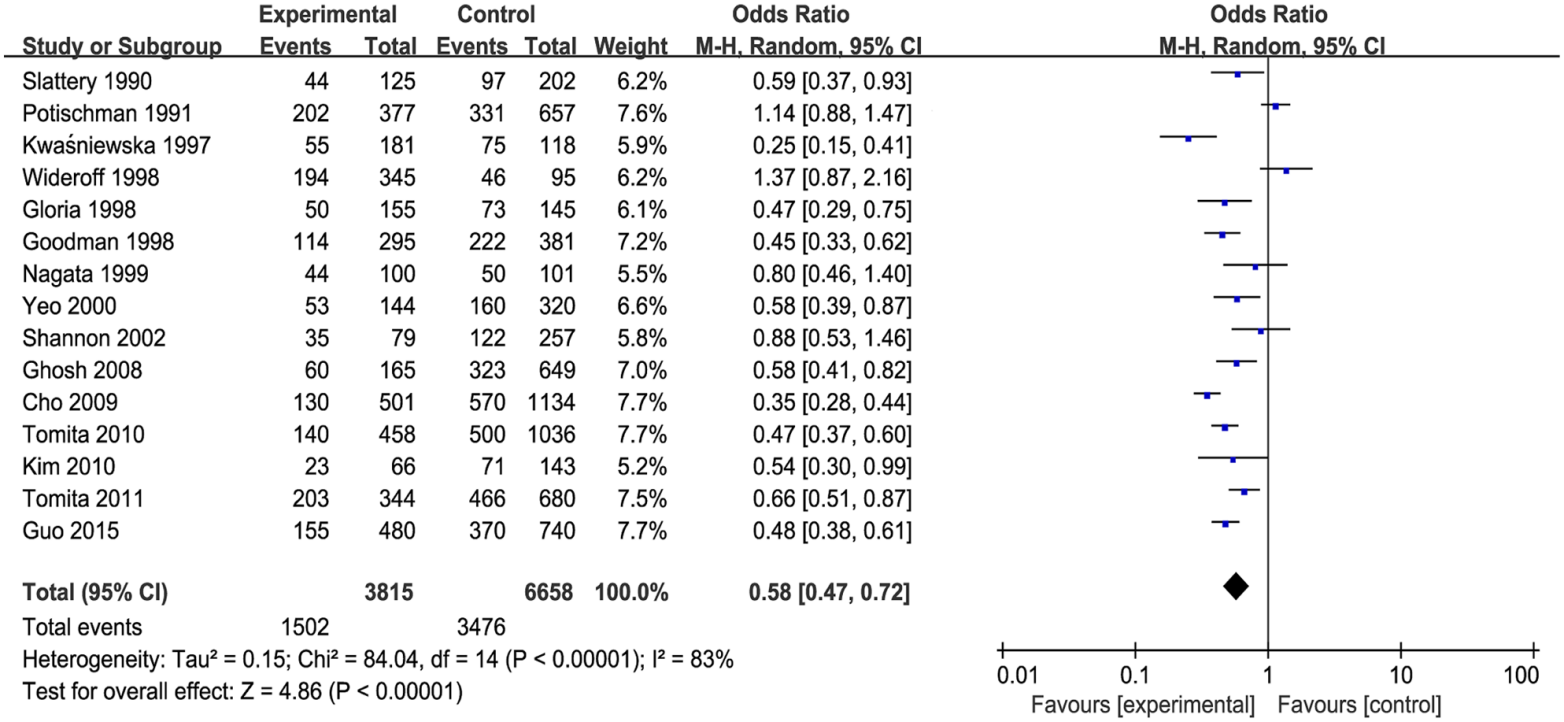
The forest plot between highest versus lowest categories of vitamin E and cervical cancer.

The pooled effect estimate was significant shown in Fig 2, which suggested that vitamin E was inversely associated with the risk of cervical neoplasia (OR = 0.58, 95% CIs = 0.47–0.72). And heterogeneity was significant (*P* < 0.00001) and considerable (*I*^2^ = 83%). In addition, both vitamin E intake and blood levels of vitamin E were negatively correlated with cervical neoplasia risk.

### Subgroup analyses and sensitivity analysis

**Fig 3:**
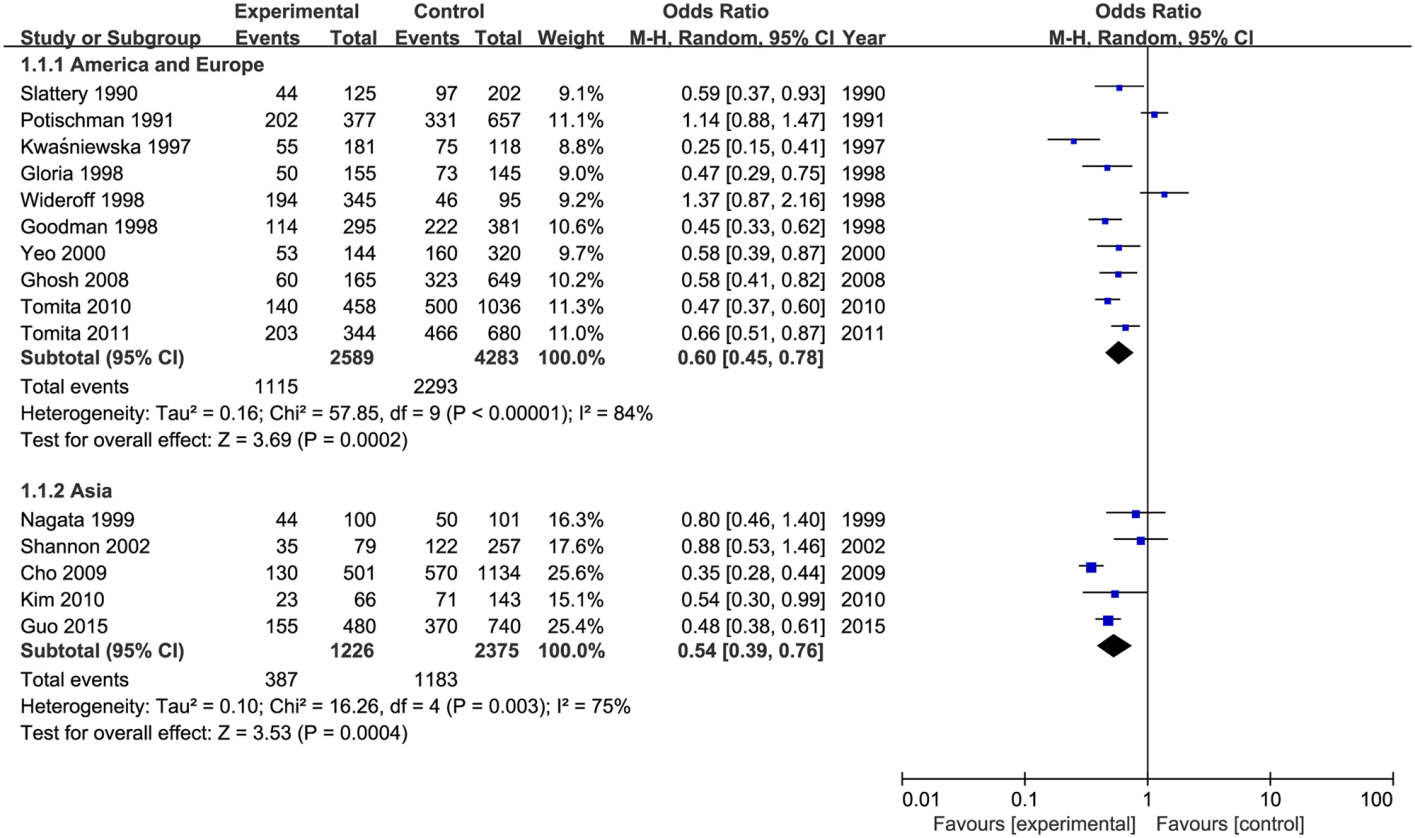
Subgroup analysis of vitamin E and cervical neoplasia in different survey ways.

**Fig 4:**
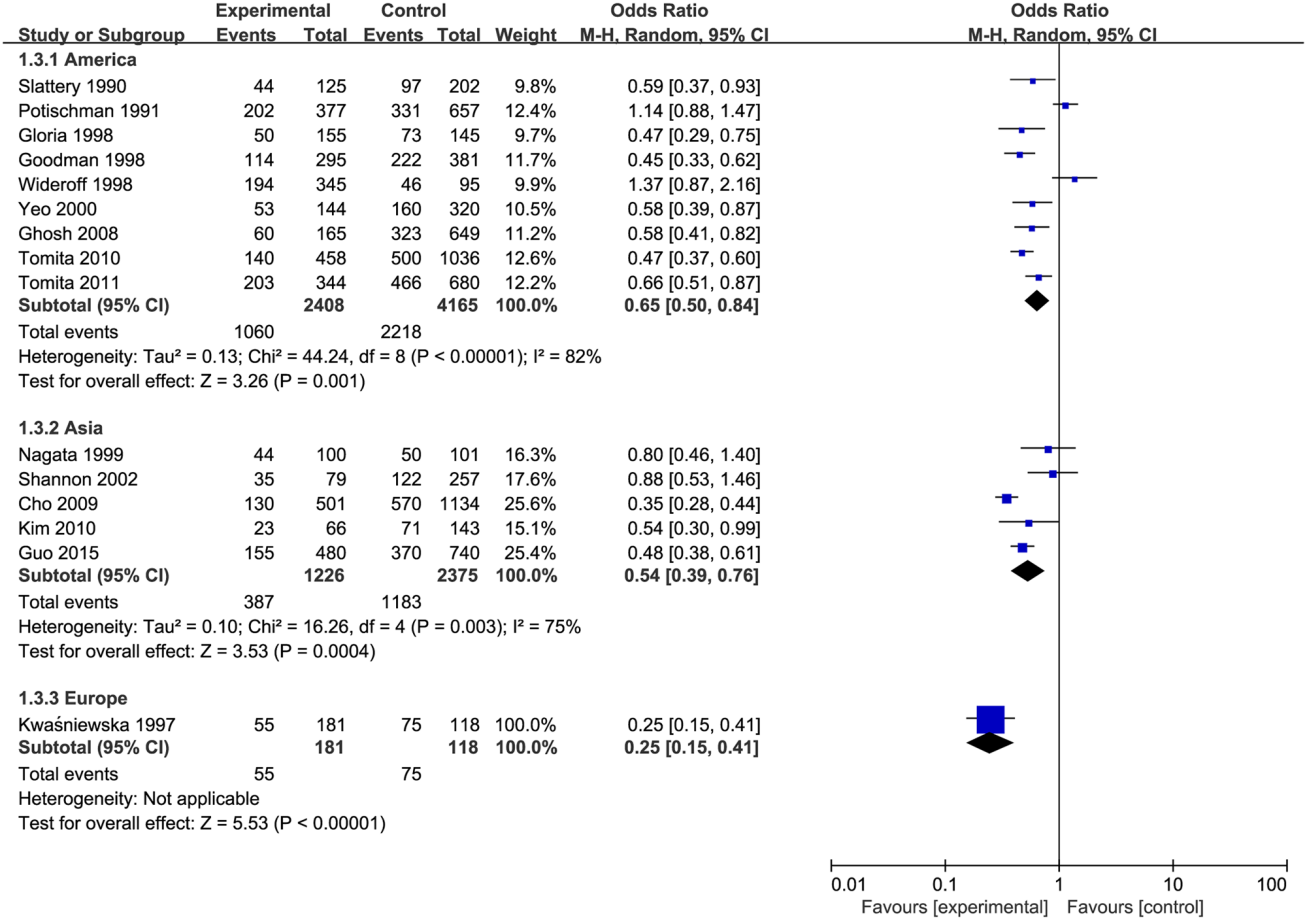
Subgroup analysis of vitamin E and cervical neoplasia in different populations.

**Fig 5:**
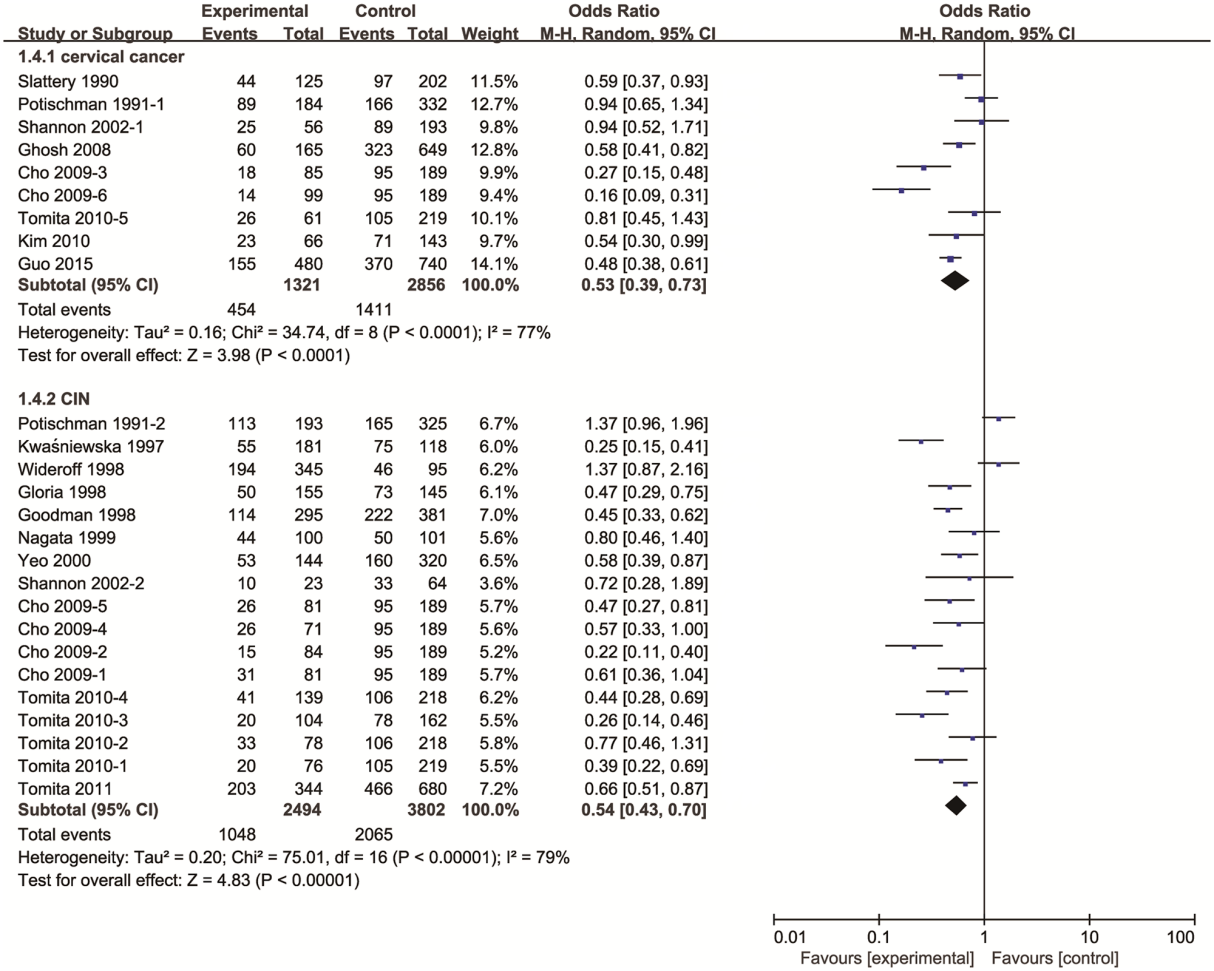
Subgroup analysis of vitamin E and different types of cervical neoplasia.

Results of subgroup analysis were reported in Table 2. The pooled ORs of cervical neoplasia shown in Fig 3 were 0.68 (95% CIs: 0.49 to 0.94, *P*_heterogeneity_ = 0.005, *I*^2^ = 70%) for vitamin E intake and 0.52 (95% CIs: 0.40 to 0.69, *P*_heterogeneity_ < 0.00001, *I*^2^ = 86%) for blood levels of vitamin E. When stratified by geographical area as shown in Fig 4, the studies from America and Europe (OR = 0.60, 95% CIs = 0.45–0.78, *P*_heterogeneity_ < 0.00001, *I*^2^ = 84%), and Asia (OR = 0.54, 95% CIs = 0.39–0.76, *P*_heterogeneity_ = 0.003, *I*^2^ = 75%) indicated that vitamin E have a significant inverse association with the risk of cervical neoplasia. As shown in Fig 5, subgroup analysis stratified by different types of cervical neoplasm indicated that the highest intake (or serum level) of vitamin E could decrease the risk for both cervical cancer (OR = 0.53, 95% CIs = 0.39–0.73, *P*_heterogeneity_ < 0.00001, *I*^2^ = 77%), and CIN (OR = 0.54, 95% CIs = 0.43–0.70, *P*_heterogeneity_ <0.00001, *I*^2^ = 79%). Meanwhile, we used sensitivity analysis to assess the influence of each single study on the pooled ORs by omitting a research in each turn. Overall, the combined ORs were not substantially different, indicating that the results of this meta-analysis were stable and reliable.

**Table 2:** Summary OR of cervical neoplasia for the highest compared with lowest of vitamin E.

Sub-groups	N	OR (95% CIs)	Heterogeneity	
			*I*^2^ (%)	*P*
Model				
Random-model	15	0.58 (0.47–0.72)	83	<0.00001
Fixed-model	15	0.56 (0.51–0.61)	83	<0.00001
**Survey ways**				
Vitamin E intake	6	0.68 (0.49–0.94)	70	= 0.005
Blood vitamin E	10	0.52 (0.40–0.69)	86	<0.00001
**Geographic locations**				
America and Europe	10	0.60 (0.45–0.78)	84	<0.00001
Asia	5	0.54 (0.39–0.76)	75	= 0.003
**Type of cervical neoplasia**				
Cervical cancer	9	0.53 (0.39–0.73)	77	<0.0001
CIN	17	0.54 (0.43–0.70)	79	<0.00001

OR, odd ratio; CIs, confidence intervals; N, number of studies; *P*, p-value for heterogeneity tests; CIN, cervical intraepithelial neoplasia.

### Assessment of heterogeneity

Heterogeneity between the included researches was indicated when the p-value for Cochran's Q test was ≤ 0.1 and the *I*^2^ value was > 50%. The related p-value of heterogeneity were listed in Table 2. Subgroup analysis and sensitivity analysis were used to explore the sources of heterogeneity between studies. However, there was still moderate to large between-study heterogeneity among the different investigations. Furthermore, meta-regression including covariates in survey ways and countries was conducted to investigate the possible sources of heterogeneity. The result of meta-regression showed that the above variables were not the sources of heterogeneity in this meta-analysis, because all of p-value were lager than 0.05 (p-value for survey ways = 0.419, p-value for geographic locations = 0.764).

### Publication bias

**Fig 6:**
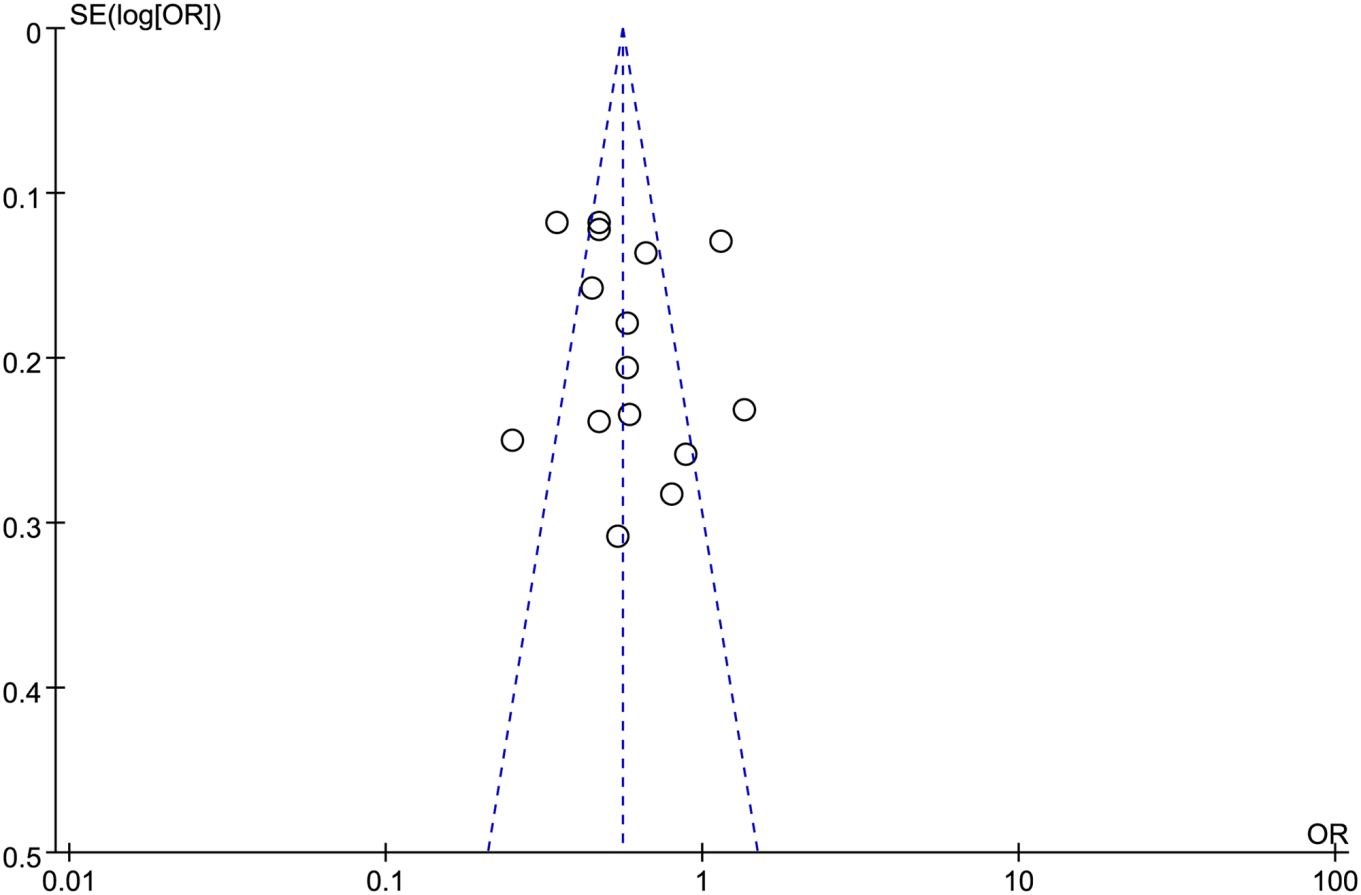
Funnel plot of vitamin E and cervical neoplasia risk.

As shown in Fig 6, the funnel plot appeared to be symmetrical, which indicated there was no obvious publication bias. However, a review of the funnel plots could not rule out the potential for publication bias for cervical neoplasia. Therefore, the Egger’s test and Begg’s test were used to detect publication bias. Additionally, there was no evidence of bias from small study effects (*P* value for Egger = 0.53; p-value for Begg = 0.322).

## Discussion

Our meta-analysis indicated that there was a significant negative relationship between vitamin E and the risk of cervical neoplasia. That is, a high level of vitamin E was significantly associated with a decreased risk of cervical neoplasia.

The incidence of several human cancers has been reported to be decreased in patients with increased dietary intake of vitamin E [27–31]. Moreover, reduced serum levels of vitamin E have been found to be associated with higher risk of several cancers, such as prostate [32–33] and breast cancers [34]. For cervical neoplasia, Srivastava et al. [35] indicated that the patients in cervical cancer had low level of antioxidant vitamin E. In a prospective Finnish study, a low serum alpha-tocopherol level was a risk factor for invasive cervical cancer [36]. However, the other studies revealed that there was different connection between vitamin E and the risk of cervical neoplasia. Lee et al. [37] concluded that there were similar plasma concentrations of alpha-tocopherol between the patients with cervical intraepithelial neoplasia and the controls. Therefore, we have systemically performed a meta-analysis to assess the relationship between vitamin E and the risk of cervical neoplasia based on the ORs for the highest versus lowest categories. We found a significant negative relationship between vitamin E and the risk of cervical neoplasia, both vitamin E intake and blood levels of vitamin E.

Nevertheless, the mechanisms of vitamin E impacted on cancer risk were still not well understood. The main mechanism of vitamin E might be as an antioxidant and anti-carcinogen, which can prevent DNA damage by scavenging lipid hydrogen peroxide radicals and finishing the lipid peroxidation chain reaction [38]. In addition, vitamin E could activate apoptosis by inhibiting the protein kinase C (PKC) pathway [39], promote immune system function [40] and restrain cancer cell growth by decreasing the phosphoinositide 3-kinase pathway [41].

While Myung et al. [42] has suggested in a previous meta-analysis that vitamin or antioxidant intake reduced the risk of cervical neoplasm, the included researches were all before 2008. Meanwhile, the article researched by Wideroff et al. [23] in 1998 was not included into the former meta-analysis. The study showed that there was not protective association between vitamin E and cervical neoplasm which was inconsistent with the result of meta-analysis. In addition, it was known that a meta-analysis was a comprehensive validation approach by analyzing the related researches in recent years, and far surpassed the evidence provided from any one study. Our study was the most up-to-date comprehensive review of vitamin E on cervical neoplasm, which summarized the updated evidence from published epidemiological researches through December 2016 and included more 6 studies than before. Therefore, the present meta-analysis included a larger number of articles and participants, which allowed a much greater possibility of reaching reliable conclusions about the association between vitamin E intake and cervical neoplasm. The present meta-analysis indicated that a high intake of vitamin E might have a protective effect against cervical neoplasia.

Meta-analysis is an important tool for indicating trends that might not be evident in a single study. There were several strengths of this meta-analysis. Sensitivity analysis suggested that the combined ORs were not altered by removing a single study each time, indicating that the results were stable. To evaluate the publication bias, the funnel plot was performed. The results of the funnel plot revealed that no publication bias existed. Our research including 15 observational studies with 3741 cases and 6328 controls obviously increased the statistical power and found a more credible relationship between vitamin E and the risk of cervical neoplasia. Between-study heterogeneity was often a concern in meta-analysis [43], and our heterogeneity test showed high heterogeneity (*I*^2^ = 83%) in the research. Therefore, the random-effects model and sensitivity analysis were used in the meta analysis. Influence analysis indicated that no individual research had excessive influence on the association of vitamin E and cervical neoplasia risk. Meanwhile, we further conducted subgroup analyses based on survey ways, geographical area and different types of cervical neoplasia to explore the source of heterogeneity. As a result, the negative relationship between vitamin E and cervical neoplasia also existed in different measuring methods, ethnicities and types of cervical neoplasia. However, moderate or high heterogeneity was present in these subgroups, indicating that other unknown confounding factors may be subsistent. Additionally, adjustments for covariates were different among these researches.

However, our study also had few limitations. First, the vitamin E formulation and dosage varied across the articles. In addition, the included studies were almost based on case-control studies. Although a case-control research can result in a recall or selection bias, such study was an important method in the study of etiology. So it was possible that our results had recall or selection bias. Thirdly, there was a possibility that the evaluation of dietary intake was mainly based on intake amounts during the past weeks which may not have precisely reflected nutrient intakes. Finally, misclassification and imprecise measurement of vitamin E intake should be of concern in observational researches.

In addition, there was also considerable heterogeneity among included researches. In the presence of heterogeneity, the application of fixed effects was inappropriate. Therefore, fixed-effects models were replaced by random-effects models in statistical analysis. Meanwhile, we didn’t find the source of heterogeneity in meta-analysis by subgroup analyses and sensitivity analysis. Furthermore, some possible sources of heterogeneity were investigated by meta-regression analysis. However, the meta-regression showed that none of the considered covariates were significantly related to the pooled effect-estimate. Therefore, despite the usage of subgroup analysis and meta-regression, the heterogeneity between studies could not be investigated completely, which might result in bias of the outcome. The possible reasons for heterogeneity were that included studies differed in various study variables, such as different regions, ethnicities, testing methodologies used, and time periods to assess the level of vitamin E. In all, heterogeneity was an important aspect in meta-analysis, and should not be ignored.

In summary, this meta-analysis suggested that both vitamin E intake and circulating vitamin E levels could reduce cervical neoplasia risk, including cervical cancer and cervical intraepithelial neoplasia. In other words, sufficient supplementation of vitamin E might reduce the risk of cervical neoplasia. However, more randomized controlled trials and cohort studies with high quality were required to further validate this inverse relationship.

## Supporting information

S1 Table PRISMA 2009 checklist. (DOC)

S2 Table Search strategy. (DOCX)

S3 Table Methodological quality of the studies. (DOCX)
