# Supplementary material for: Effect of vitamin E supplementation on uterine cervical neoplasm: A meta-analysis of case-control studies
Source: PLoS One. 2017 Aug 22;12(8):e0183395. doi: 10.1371/journal.pone.0183395 (PMC5567498; doi:10.1371/journal.pone.0183395)
Supplement: S2 Table — (DOCX) [file pone.0183395.s002.docx]

**Table S2. Search Strings for Vitamin E and Uterine cervical neoplasm in Pubmed. *** Notes the MESH term used in the database.

| **Search Strings AND** | |
| --- | --- |
| Vitamin E* | Uterine Cervical Neoplasms* |
| Vit E | Cervical cancer |
| tocopherols | Cervical tumor |
| alpha-tocopherol | Cervical malignance |
| antioxidant | Cervical carcinoma |
| diet | Cervical neoplasm  Cervical Intraepithelial Neoplasia* |
