# Supplementary material for: Effect of vitamin E supplementation on uterine cervical neoplasm: A meta-analysis of case-control studies
Source: PLoS One. 2017 Aug 22;12(8):e0183395. doi: 10.1371/journal.pone.0183395 (PMC5567498; doi:10.1371/journal.pone.0183395)
Supplement: S3 Table — (DOCX) [file pone.0183395.s003.docx]

**Table S3. Methodological quality of the studies included in the meta-analysis.**

| study | adequate definition of cases | Representativeness of the cases | Selection of Controls | Definition of Controls | Control for important factor or additional factor | Ascertainment of exposure | Same method to ascertain for cases and controls | Non-  Response rate | scores |
| --- | --- | --- | --- | --- | --- | --- | --- | --- | --- |
| Gloria | ★ | ★ | ★ | ★ | ★- | ★ | ★ | - | 7 |
| Kim | ★ | ★ | ★ | ★ | ★- | ★ | ★ | ★ | 8 |
| Nagata | ★ | ★ | ★ | - | ★- | ★ | ★ | - | 6 |
| Potischman | ★ | ★ | ★ | - | ★- | ★ | ★ | - | 6 |
| Tomita2010 | ★ | ★ | ★ | ★ | ★- | ★ | ★ | ★ | 8 |
| Tomita2011 | ★ | ★ | ★ | ★ | ★- | ★ | ★ | - | 7 |
| Yeo | ★ | ★ | ★ | ★ | ★- | ★ | ★ | - | 7 |
| Cho | ★ | ★ | ★ | ★ | ★- | ★ | ★ | ★ | 8 |
| Goodman | ★ | ★ | ★ | ★ | ★- | ★ | ★ | - | 7 |
| Guo | ★ | ★ | ★ | ★ | ★- | ★ | ★ | ★ | 8 |
| Kwaśniewska | ★ | ★ | ★ | ★ | - | ★ | ★ | - | 6 |
| Shannon | ★ | ★ | ★ | ★ | ★- | ★ | ★ | - | 7 |
| Wideroff | ★ | ★ | ★ | ★ | ★- | ★ | ★ | - | 7 |
| Ghosh | ★ | ★ | ★ | ★ | ★- | ★ | ★ | ★ | 8 |
| Slattery | ★ | ★ | ★ | ★ | ★- | ★ | ★ | - | 7 |
